# Nanoparticle-Mediated Drug Delivery System for Pulmonary Arterial Hypertension

**DOI:** 10.3390/jcm6050048

**Published:** 2017-04-29

**Authors:** Kazufumi Nakamura, Hiromi Matsubara, Satoshi Akagi, Toshihiro Sarashina, Kentaro Ejiri, Norifumi Kawakita, Masashi Yoshida, Toru Miyoshi, Atsuyuki Watanabe, Nobuhiro Nishii, Hiroshi Ito

**Affiliations:** 1 Department of Cardiovascular Medicine, Okayama University Graduate School of Medicine, Dentistry and Pharmaceutical Sciences, 2-5-1 Shikata-cho, Kita-ku, Okayama 700-8558, Japan; akagi-s@cc.okayama-u.ac.jp (S.A.); sarashina.toshihiro@gmail.com (T.S.); eziken82@gmail.com (K.E.); rm3ta6@bma.biglobe.ne.jp (N.K.); masashiyoshid@gmail.com (M.Y.); miyoshit@cc.okayama-u.ac.jp (T.M.); atsunao0422@gmail.com (A.W.); nnnnishii2001@gmail.com (N.N.); itomd@md.okayama-u.ac.jp (H.I.); 2 Division of Cardiology, National Hospital Organization Okayama Medical Center, Okayama 701-1192, Japan; matsubara.hiromi@gmail.com

**Keywords:** pulmonary arterial hypertension, prostacyclin, nanoparticle, drug delivery system

## Abstract

Nanoparticles have been used as a novel drug delivery system. Drug-incorporated nanoparticles for local delivery might optimize the efficacy and minimize the side effects of drugs. The efficacy and safety of intratracheal administration of prostacyclin analog (beraprost) -incorporated nanoparticles and imatinib (a PDGF-receptor tyrosine kinase inhibitor) -incorporated nanoparticles in Sugen-hypoxia-normoxia or monocrotaline rat models of pulmonary arterial hypertension (PAH) and in human PAH-pulmonary arterial smooth muscle cells have been reported. The use of inhaled drug-incorporated nanoparticles might be a novel approach for the treatment of PAH.

## 1. Introduction

Pulmonary arterial hypertension (PAH) is a life-threatening and progressive disease characterized by progressively elevated pulmonary vascular resistance (PVR) and pulmonary artery pressure. Increased PVR is induced by pulmonary vasoconstriction, vascular remodeling by intimal and medial hypertrophy, and thrombosis [1,2,3]. Sustained elevation of pulmonary vascular resistance causes severe right ventricular (RV) hypertrophy and failure, leading to a poor prognosis. PAH-targeted drugs, including prostacyclin (prostaglandin I_2_), endothelin receptor antagonists (ERAs), phosphodiesterase type-5 inhibitors (PDE-5i) and a soluble guanylate cyclase stimulator, have become available in the past two decades, and treatment with these vasodilators has been effective [4,5,6,7,8]. However, their full therapeutic abilities are reduced by medication non-compliance and side effects, and PAH is still a fatal disorder in many patients. To solve these problems, several novel therapeutic strategies for PAH, including nanoparticle-mediated drug delivery systems, (nano-DDS) are proposed.

## 2. Prostacyclin Therapy for PAH

The release of endogenous prostacyclin (prostaglandin I_2_) is depressed in patients with PAH. Prostacyclin replacement therapy by infusion of epoprostenol sodium, a prostacyclin (prostaglandin I_2_), is one of the best treatments available for PAH. High-dose epoprostenol therapy (>40 ng/kg/min) resulted in marked hemodynamic improvement in patients with idiopathic PAH (IPAH) [4,9]. Compared with the baseline state, high-dose epoprostenol therapy reduced mean pulmonary arterial pressure (mPAP) by 30% and PVR by 68%. We have also reported that high-dose epoprostenol has a pro-apoptotic effect on pulmonary artery smooth muscle cells (PASMCs) of patients with PAH via the IP receptor [10].

However, epoprostenol therapy causes several adverse events and complications such as headaches, hypotension and catheter-related infections. Chronic infusion of epoprostenol is performed using a small, portable infusion pump through an indwelling central venous catheter. The most serious complication is catheter-related infections. The catheter infection rate was 0.26 per 1000 catheter days in PAH patients treated with epoprostenol [11]. Systemic administration of prostacyclin can induce headaches, flushing and sometimes severe hypotension at the start of prostacyclin therapy. These problems would be solved if an alternative system that targeted the delivery of the prostacyclin to the pulmonary vasculature without using a central venous catheter is developed.

## 3. Imatinib for the Treatment of PAH

Remodeling of the pulmonary artery by an inappropriate increase of PASMCs is problematic in the treatment of PAH. Effective treatment that achieves reverse remodeling is required. This will require anti-proliferative and pro-apoptotic agents for PASMCs.

We have reported that platelet-derived growth factor (PDGF)-BB stimulation caused a higher growth rate of cultured PASMCs from patients with IPAH than that of control cells [12,13]. Imatinib is a PDGF-receptor tyrosine kinase inhibitor, and is a drug used to treat certain types of cancer such as chronic myelogenous leukemia and gastrointestinal stromal tumors. Schermuly et al. reported that imatinib reverses pulmonary vascular remodeling and cor pulmonale in rats with monocrotaline-induced pulmonary hypertension (PH), as well as in mice with chronic hypoxia-induced PH [14]. Imatinib has anti-proliferative and pro-apoptotic effects on IPAH-PASMCs stimulated with (PDGF)-BB [15].

Clinical improvement and hemodynamic improvement have been reported in some patients with PAH who were treated with imatinib [16,17]. However, a randomized, double-blind, placebo-controlled trial showed that imatinib improved exercise capacity and hemodynamics in patients with severe PAH, but that serious adverse events and drug discontinuations were common with this treatment [18]. Since systemic administration of imatinib causes serious adverse events, the development of a new route of administration is required.

## 4. Nano-DDS

Nanoparticles (NPs) have been used as a novel delivery system for the transport of drugs to target organs [19,20,21]. NPs are taken up by the target organ because of their small size, permeability, and retention effect. Drug release from NPs is controlled according to the NP composition. Thus, drug-incorporated NPs for local delivery might optimize the efficacy and minimize the side effects of drugs. Liposomes and polymers have been tested as nano-DDSs in basic and clinical studies.

As for the treatment of PAH, treatment with vasodilators such as prostacyclin, ERAs and PDE-5i has been effective [4,5,6,7,8], though PAH is still a fatal disorder in many patients. Several novel therapeutic strategies for PAH including nano-DDS are proposed. Systemic administration of prostacyclin or imatinib causes several adverse events and complications. Nano-DDSs targeting the lung would optimize the efficacy and minimize the side effects of drugs. Pitavastatin [22], nuclear factor kappaB decoy [23], imatinib [24], prostacyclin analog [25,26], fasudil [27], and antimiRNA-145-incorporated NPs [28] have been reported to be effective in animal models of PAH (Table 1). In this review, we summarize the results of these studies. All of the data in these studies were obtained in rat models of PAH and have not yet been translated to human trials. The efficacy and safety of nano-DDSs for PAH in humans are unknown. Further studies are needed to clarify these points.

### 4.1. Prostacyclin Analog-Incorporated NPs

**Figure 1:**
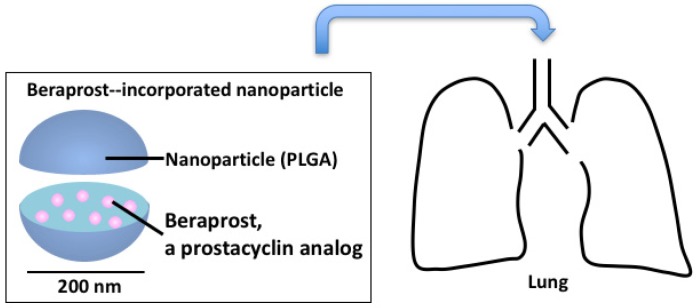
Prostacyclin analog-incorporated nanoparticles for treatment of pulmonary arterial hypertension.

We investigated the efficacy and safety of intratracheal administration of NPs incorporated with beraprost, a prostacyclin analog (beraprost-NPs) in Sugen-hypoxia-normoxia and monocrotaline (MCT) rat models of PAH (Figure 1) [26]. Nanoparticles of polylactide-glycolide (PLGA), a polymer, encapsulated with beraprost were used in the study. After a single administration, beraprost-NPs significantly decreased right ventricular pressure, right ventricular hypertrophy, and pulmonary artery muscularization in both rat models. Beraprost-NPs significantly improved the survival rate in the MCT rat model. No infiltration of inflammatory cells, hemorrhage, or fibrosis was found in the liver, kidney, spleen or heart after the administration of beraprost-NPs.

Ishihara et al. also reported that the intravenous administration of beraprost encapsulated into nanoparticles prepared from a poly(lactide) homopolymer (PLA) and monomethoxy poly(ethyleneglycol)-poly(lactide) block copolymer protected against MCT-induced pulmonary arterial remodeling and right ventricular hypertrophy, and that once per week intravenous administration of beraprost-NPs also had an ameliorative effect on hypoxia-induced pulmonary arterial remodeling and right ventricular hypertrophy [25].

### 4.2. Imatinib-Incorporated NPs

We examined the efficacy of imatinib-incorporated NPs (Ima-NPs) in the MCT rat model of PAH and in human PAH-PASMCs [24]. A single intratracheal administration of Ima-NPs suppressed the development of MCT-induced PAH in rats. Ima-NPs had sustained antiproliferative effects on human PAH-PASMCs.

### 4.3. Pitavastatin-Incorporated NPs

Chen et al. reported that intratracheal treatment with pitavastatin-incorporated NP, but not with pitavastatin, attenuated the development of MCT-induced PAH in rats and was associated with a reduction of inflammation and pulmonary arterial remodeling [22]. The fluorescein isothiocyanate (FITC) signals were detected in small bronchial tracts, alveolar macrophages, and small pulmonary arteries (PAs) for up to 14 days after a single instillation of FITC-encapsulated PLGA NP. Furthermore, treatment with pitavastatin-NP three weeks after MCT injection induced the regression of PAH and improved the survival rate. These results suggest potential clinical significance for developing a new treatment for PAH.

### 4.4. Fasudil-Incorporated NPs

Pulmonary hypertension is associated with hypoxic exposure, endothelial dysfunction, and PASMC hypercontraction and proliferation for which Rho-kinase seems to be substantially involved [29]. Long-term treatment with fasudil, a Rho-kinase inhibitor, suppresses the development of MCT-induced PAH in rats [30] and hypoxia-induced PAH in mice [31].

Gupta et al. reported that intratracheal instillation of liposomal fasudil reduced mPAP in MCT-induced PAH rats and that the effect continued for about 3 h, suggesting that liposomal formulations produced pulmonary preferential vasodilation [27].

### 4.5. Oligonucleotides-Incorporated NPs

Decoy oligonucleotides are used as inhibitors of specific gene expression without any changes in the functions of other genes [32]. Kimura et al. reported that the activity of nuclear factor *k*B (NF-kB), a transcription factor which regulates proinflammatory cytokines, increased in the MCT rat model of PAH, while intratracheal instillation of NF-kB decoy oligonucleotides-NPs (PEG-PLGA) attenuated the development of MCT-induced PAH in rats [23]. Moreover, treatment with NF-kB decoy NPs three weeks after MCT injection improved the survival rate as compared with vehicle administration.

RNA interference (RNAi) reduces the expression of pathological proteins. Currently, RNAi-based therapies exhibit the potential to be a novel therapeutic strategy in various diseases [33]. McLendon et al. reported that lipid nanoparticle delivery of an antisense oligonucleotide against microRNA-145 improved Sugen-hypoxia-induced PAH in rats [28].

## 5. Summary and Clinical Perspective

In this review, we summarized the properties of selected nano-DDSs and the results of preclinical studies using the nano-DDSs in animal models of PAH. Drug-incorporated nanoparticles for local delivery might optimize the efficacy and minimize the side effects of drugs.

A phase I investigator-initiated clinical trial to test the efficacy of PLGA nanoparticle-mediated delivery of pitavastatin (UMIN000014940) has been completed. Future clinical trials may prove the safety and efficacy of a nano-DDS for PAH.

## Figures and Tables

**Table 1:** Nanoparticle-mediated drug delivery systems for pulmonary arterial hypertension treatment.

Drug	Delivery System	Animal Model	Route of Administration	References
Pitavastatin	Polymer (PLGA)	MCT-induced rat model	Intratracheal	[22]
NF-kB decoy	Polymer (PEG-PLGA)	MCT-induced rat model	Intratracheal	[23]
Imatinib	Polymer (PLGA)	MCT-induced rat model	Intratracheal	[24]
Beraprost	Polymer (PLA and PEG-PLA)	MCT-induced rat model	Intravenous	[25]
Beraprost	Polymer (PLGA)	MCT-induced rat model	Intratracheal	[26]
		Sugen/hypoxia rat model		
Fasudil	Liposome	MCT-induced rat model	Inhalation	[27]
AntimiRNA-145	Liposome	Sugen/hypoxia rat model	Intravenous	[28]

Note: NF-kB, nuclear factor kappaB; PLGA, polylactide-glycolide; PEG-PLGA, poly-(ethyleneglycol)-block-PLGA; MCT, monocrotaline; PLA, poly(lactide) homoplymer; PEG-PLA, poly-(ethyleneglycol)-PLA.
